# Predicting protein-protein interactions via multivariate mutual information of protein sequences

**DOI:** 10.1186/s12859-016-1253-9

**Published:** 2016-09-27

**Authors:** Yijie Ding, Jijun Tang, Fei Guo

**Affiliations:** 1 School of Computer Science and Technology, Tianjin University, No.135, Yaguan Road, Tianjin Haihe Education Park, Tianjin, People’s Republic of China; 2 Department of Computer Science and Engineering, University of South Carolina, Columbia, USA

**Keywords:** Protein-protein interactions, Protein sequence, Feature extraction, Conjoint amino acids, Multivariate mutual information

## Abstract

**Background:**

Protein-protein interactions (PPIs) are central to a lot of biological processes. Many algorithms and methods have been developed to predict PPIs and protein interaction networks. However, the application of most existing methods is limited since they are difficult to compute and rely on a large number of homologous proteins and interaction marks of protein partners. In this paper, we propose a novel sequence-based approach with multivariate mutual information (MMI) of protein feature representation, for predicting PPIs via Random Forest (RF).

**Methods:**

Our method constructs a 638-dimentional vector to represent each pair of proteins. First, we cluster twenty standard amino acids into seven function groups and transform protein sequences into encoding sequences. Then, we use a novel multivariate mutual information feature representation scheme, combined with normalized Moreau-Broto Autocorrelation, to extract features from protein sequence information. Finally, we feed the feature vectors into a Random Forest model to distinguish interaction pairs from non-interaction pairs.

**Results:**

To evaluate the performance of our new method, we conduct several comprehensive tests for predicting PPIs. Experiments show that our method achieves better results than other outstanding methods for sequence-based PPIs prediction. Our method is applied to the *S*.*cerevisiae* PPIs dataset, and achieves 95.01 % accuracy and 92.67 % sensitivity repectively. For the *H*.*pylori* PPIs dataset, our method achieves 87.59 % accuracy and 86.81 % sensitivity respectively. In addition, we test our method on other three important PPIs networks: the one-core network, the multiple-core network, and the crossover network.

**Conclusions:**

Compared to the Conjoint Triad method, accuracies of our method are increased by 6.25,2.06 and 18.75 %, respectively. Our proposed method is a useful tool for future proteomics studies.

## Background

Identification of protein-protein interactions (PPIs) is important to elucidate protein functions and identify biological processes in a cell. The knowledge of PPIs can help people better understand disease mechanisms and drug designs. In the past several years, a large number of technologies have been developed for the large-scale analysis of PPIs. In general, there are three categories of methods for detecting PPIs: methods based on the information of evolution, methods based on natural language processing, and methods based on features of amino acid sequence.

A large number of past studies have made clear that the protein-protein interaction has a co-evolution trend [1]. The evolution information is extracted from multiple sequence alignment of homologous proteins. Tree similarity is used as a simple linear correlation between distance matrices of two protein families, as a proxy of their phylogenetic trees [2]. MirrorTree [3–5] evaluates the relationship between tree similarities and physical or functional interactions. It is possible to predict PPIs on a genomic scale with higher correlations indicating a higher probability of protein-protein interaction. Carlo et al. [6] presented a log-likelihood score for protein-protein interaction. Direct Coupling Analysis (DCA) has been used to predict response regulator (RR) interaction partners for orphan histidine sensor kinase (SK) proteins in bacterial two-component signal transduction systems [7]. They also presented a protein-protein interaction score, which is based on improved efficiency of multivariate gaussian approach [8]. However, since these methods need a large number of homologous proteins and interaction marks of protein partners, they are very difficult to compute and their applications are limited.

Many methods have been developed to find the evidence for PPIs from PubMed abstracts based on Natural Language Processing (NLP) [9]. According to a certain semantic model, these methods automatically extract relevant pieces of information from texts, since a large number of known PPIs are stored in the scientific literature of biology and medicine. Daraselia et al. [10] used a method, called MedScan, to extract more than one million pieces of data from PubMed. They obtained accuracy rates of up to 91 %, compared with the BIND and DIP databases [11]. The problem of this approach is that some PPIs information may be missing from literature, thus the prediction may not be complete.

It might be possible to predict PPIs accurately by using only protein sequence information with methods based on machine learning algorithms and features of amino acids. To use machine learning methods in this task, one of the most important computational challenges is to extract useful features from protein sequences. Generally, there are several kinds of feature representation methods including Auto Covariance (AC) [12], Auto Cross Covariance (ACC) [12], Conjoint Triad (CT) [13], Local Protein Sequence Descriptors (LD) [14, 15], Multi-scale Continuous and Discontinuous feature set(MCD) [16], Physicochemical Property Response Matrix combined with Local Phase Quantization descriptor (PR-LPQ) [17], Multi-scale Local Feature Descriptors (MLD) [18], as well as Substitution Matrix Representation (SMR) [19].

AC and ACC [12] use seven physicochemical properties of amino acids to reflect their interaction modes whenever possible. After being represented by these seven descriptors, a pair of proteins could be converted into a 420-dimensional vector by AC, and 2940-dimension by ACC. CT [13] considers the properties of each amino acid and its vicinal neighbors and regards the three contiguous amino acids as a unit. The PPIs information of protein sequences can be projected into a homogeneous vector space by counting the frequency of each type. The 20 amino acids are clustered into seven groups according to dipoles and volumes of side chains. The descriptor of proteins were concatenated into a 686-dimensional vector by CT.

Similar to CT, LD [14, 15] clusters twenty standard amino acids into seven functional groups. It splits the protein sequence into ten local regions of varying length to describe multiple overlapping continuous and discontinuous interaction patterns within a protein sequence. For each local region, three local descriptors–composition (C), transition (T) and distribution (D)–are calculated. A 1260-dimentional vector is constructed to represent each protein pair by LD. MLD [18] uses a multi-scale decomposition technique to divide protein sequence into multiple sequence segments of varying length to describe overlapping local regions. A binary coding scheme is then adopted to construct a set of continuous regions on the basis of the above partition. A 1134-dimentional vector is constructed to represent each protein pair by MLD. MCD [16] is similar to MLD, except that it constructs a 1764-dimentional vector for each protein pair. Indeed, LD, MCD and MLD can be categorized as the same type of methods.

PR-LPQ [17] adopts the physicochemical property response matrix method to transform the amino acids sequence into a matrix and then employs the local phase quantization-based texture descriptor to extract local phrase information in the matrix. SMR is based on BLOSUM62, which is considered to be powerful for detecting weak protein similarities. Huang et al. [19] used BLOSUM62 to construct a new matrix representation from a protein sequence. Then, the matrix is lossy compressed by Discrete Cosine Transform(DCT) and a 400-dimensional feature vector is extracted from the compressed matrix. Each pair of protein sequences forms an 800-dimensional feature vector, which is fed into the Weighted Sparse Representation based Classifier(WSRC) for predicting PPIs.

In this paper, we propose a novel sequence-based approach with a *k*-gram feature representation calculated as Multivariate Mutual Information (MMI). Combined with normalized Moreau-Broto Autocorrelation (NMBAC), we predict PPIs via Random Forest (RF), which is an ensemble learning method for classification, regression and other tasks. For the performance evaluation, our method is applied to the *S*.*cerevisiae* PPIs dataset. Our method achieves 95.01 % accuracy and 92.67 % sensitivity. Compared with the existing best method, the accuracy is increased by 0.29 %. To further demonstrate the effectiveness of our method, we also test it on the *H*.*pylori* PPIs dataset. Our method achieves 87.59 % accuracy and 86.81 % sensitivity. On the *human*_8161_ PPIs dataset, our method achieves 97.56 % accuracy and 96.57 % sensitivity. In addition, we use *S*.*cerevisiae* PPIs dataset to construct a model to predict five other independent species PPIs datasets. Compared with the state-of-the-art methods, the accuracy is increased 2.42 % on average. We also test our method on two special PPIs datasets [20]. On the yeast dataset, our method achieves 82,82,62 and 61 % AUROC on four different test classes (typical Cross-Validated (CV) and distinct test classes C1, C2 and C3). On the human dataset, our method achieves 82,82,60 and 57 % AUROC on four different test classes. Finally, we test our method on three important PPIs networks: the one-core network (CD9) [21], the multiple-core network (Ras-Raf-Mek-Erk-Elk-Srf pathway) [22], and the crossover network (Wnt-related Network) [23]. Compared to the Conjoint Triad (CT) method [13], accuracies of our method are increased by 6.25,2.06 and 18.75 %, respectively.

## Methods

In our method for predicting protein-protein interaction based on protein sequence information, first we extract features from protein sequence information. The feature vector represents the characteristic on one pair of proteins. We use *k*-gram feature representation calculated as Multivariate Mutual Information (MMI) and extract additional feature by normalized Moreau-Broto Autocorrelation (NMBAC) from protein sequences. These two approaches are employed to transform the protein sequence into feature vectors. Then, we feed the feature vectors into a specific classifier for identifying interaction pairs and non-interaction pairs.

### Multivariate mutual information

Inspired by previous work [13, 24, 25] for extracting features from protein sequences, we propose a novel method to fully describe key information of protein-protein interaction. There exist many technologies using the *k*-gram feature representation, which is commonly used for protein sequence classification [26, 27]. Here *k* represents the number of conjoint amino acids. For example, CT [13] used the 3-gram feature representation. Shen et al. [13] indicated that methods without considering local environment are usually not reliable and robust, so they produced a conjoint triad method to consider properties of amino acids and their proximate amino acids.

To continue the usage of *k*-gram feature representation and to enhance classification accuracy, we utilize MMI [28] for deeply extracting conjoint information of amino acids in protein sequences.

#### Classifying amino acids

The protein-protein interaction can be dominated by dipoles and volumes of diverse amino acids, which reflect electrostatic and hydrophobic properties. All 20 standard amino acid types are assigned to seven functional groups [13], as shown in Table 1. For each pair of proteins, we extract conjoint information based on these amino acid categories.

**Table 1:** Division of 20 amino acid types, based on dipoles and volumes of side chains

*No*.	*Group*	*Dipolescale*	*Volumescale*
*C*_0_	*A*,*G*,*V*	Dipole <1.0	Volume <50
*C*_1_	*C*	1.0< Dipole <2.0 (form disulphide bonds)	Volume >50
*C*_2_	*D*,*E*	Dipole >3.0 (opposite orientation)	Volume >50
*C*_3_	*F*,*I*,*L*,*P*	Dipole <1.0	Volume >50
*C*_4_	*H*,*N*,*Q*,*W*	2.0< dipole <3.0	Volume >50
*C*_5_	*K*,*R*	Dipole >3.0	Volume >50
*C*_6_	*M*,*S*,*T*,*Y*	1.0< dipole <2.0	Volume >50

#### Calculating multivariate mutual information

**Fig. 1:**
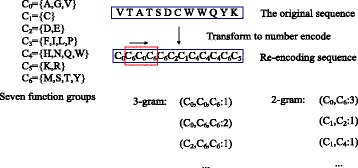
3-gram or 2-gram feature representation

Considering the neighbours of each amino acid, we regard any three contiguous amino acids as a unit. We use a sliding window of a length of 3 amino acids to parse the protein sequence. For each window, categories of three amino acids are used to label the type of this unit. Instead of considering the order of the three amino acids, we only consider the basic ingredient of the unit. We define different types of 3-gram feature representation, such as ^′^*C*_0_,*C*_0_,*C*0′,^′^*C*_0_,*C*_0_,*C*1′,…,^′^*C*_6_,*C*_6_,*C*6′. Similarly, we also define different types of 2-gram feature representation, such as ^′^*C*_0_,*C*0′,^′^*C*_0_,*C*1′,…,^′^*C*_6_,*C*6′. We count each type of 3-gram feature and 2-gram feature on one protein sequence by a sliding window, as shown in Fig. 1.

At some point in the ensuing discussion of mutual information, we state the logarithmic base as *e*. In contrast to the standard mutual information approach, our mutual information and entropy method refer to single event on one protein sequence, whereas standard mutual information refers to overall possible events. We calculate the multivariate mutual information for each type of 3-gram feature, defined as follows:

1 $$ I(a,b,c)= I(a,b)- I(a,b|c)  $$

where *a*,*b* and *c* are categories of three conjoint amino acids in one unit.

We then define the mutual information for one type of 2-gram feature as *I*(*a*,*b*), which can be counted by a 2-length sliding window:

2 $$ I(a,b) = f(a,b)ln\left(\frac{f(a,b)}{f(a)f(b)}\right)  $$

where *f*(*a*,*b*) is the frequency of categories *a* and *b* appearing in 2-gram feature on a protein, and *f*(*a*) is the frequency of category *a* appearing on a protein, respectively.

In addition, we define the conditional mutual information as *I*(*a*,*b*|*c*).

3 $$ I(a,b|c)=H(a|c)-H(a|b,c)  $$

where *H*(*a*|*c*) and *H*(*a*|*b*,*c*) are the conditional entropy as follows.

4 $$ H(a|c) = -f(a|c)ln(f(a|c))  $$

and

5 $$ H(a|b,c)=-f(a|b,c)ln(f(a|b,c))  $$

where *f*(*a*|*c*) is the frequency of category *a* appearing while category *c* exists in 2-gram feature on a protein, and *f*(*a*|*b*,*c*) is the frequency of category *a* appearing while categories *b* and *c* exist in 3-gram feature on a protein.

*H*(*a*|*c*) and *H*(*a*|*b*,*c*) can be approximately calculated as follows:

6 $$ H(a|c) = -\frac{f(a,c)}{f(c)}ln\left(\frac{f(a,c)}{f(c)}\right)  $$

and

7 $$ H(a|b,c)=-\frac{f(a,b,c)}{f(b,c)}ln\left(\frac{f(a,b,c)}{f(b,c)}\right)  $$

where *f*(*a*,*b*,*c*) is the frequency of categories *a*,*b* and *c* appearing in 3-gram feature on a protein.

To avoid the values of *I*(*a*,*b*,*c*) and *I*(*a*,*b*) being infinity, we calculate the frequency as follows:

8 $$ f(a) = \frac{n_{a} + 1}{L+1}  $$

where *n*_*a*_ is the occurrence number of category *a* appearing on a protein and *L* is the length of this protein sequence. We also use similar formulas to calculate *f*(*a*,*b*) and *f*(*a*,*b*,*c*).

We can get 84 multivariate mutual information values of *I*(*a*,*b*,*c*) (3-tuples MI) and 28 mutual information values of *I*(*a*,*b*) (2-tuples MI) from one protein. We also compute the frequency of the seven amino acid categories appearing on this protein. A protein sequence is represented as 84+28+7=119 features. Finally, we combine the descriptors of two proteins to build a 238-dimensional vector for representing each pair of proteins.

### Normalized moreau-broto autocorrelation

It is well known that PPIs include four interaction modes, usually expressed as electrostatic interaction, hydrophobic interaction, steric interaction and hydrogen bond. Feng et al. [29] introduced an autocorrelation function combining physicochemical properties of amino acids to propose a feature representation method, which is used to predict the types of membrane proteins. Inspired by this method, we use the NMBAC to extract features from protein sequences.

#### Six physicochemical properties of amino acid

The physicochemical properties we consider are hydrophobicity (H), volumes of side chains of amino acids (VSC), polarity (P1), polarizability (P2), solvent-accessible surface area (SASA) and net charge index of side chains (NCISC) of amino acid.

Values of these six physicochemical properties for each amino acid are listed in Table 2 [30]. They are first normalized to zero mean and unit standard deviation (SD) as follows:

9 $$ P_{i,j}^{'} = \frac{P_{i,j}-P_{j}}{S_{j}}(i=1,2,\ldots,20;j=1,2,\ldots,6.)  $$

**Table 2:** Original values of six physicochemical properties of 20 amino acid types

Amino acid	H	VSC	P1	P2	SASA	NCISC
A	0.62	27.5	8.1	0.046	1.181	0.007187
C	0.29	44.6	5.5	0.128	1.461	-0.03661
D	-0.9	40	13	0.105	1.587	-0.02382
E	-0.74	62	12.3	0.151	1.862	0.006802
F	1.19	115.5	5.2	0.29	2.228	0.037552
G	0.48	0	9	0	0.881	0.179052
H	-0.4	79	10.4	0.23	2.025	-0.01069
I	1.38	93.5	5.2	0.186	1.81	0.021631
K	-1.5	100	11.3	0.219	2.258	0.017708
L	1.06	93.5	4.9	0.186	1.931	0.051672
M	0.64	94.1	5.7	0.221	2.034	0.002683
N	-0.78	58.7	11.6	0.134	1.655	0.005392
P	0.12	41.9	8	0.131	1.468	0.239531
Q	-0.85	80.7	10.5	0.18	1.932	0.049211
R	-2.53	105	10.5	0.291	2.56	0.043587
S	-0.18	29.3	9.2	0.062	1.298	0.004627
T	-0.05	51.3	8.6	0.108	1.525	0.003352
V	1.08	71.5	5.9	0.14	1.645	0.057004
W	0.81	145.5	5.4	0.409	2.663	0.037977
Y	0.26	117.3	6.2	0.298	2.368	0.023599

where *P*_*i*,*j*_ is the value of descriptor *j* for amino acid type *i*,*P*_*j*_ is the mean over 20 amino acids of descriptor value *j*, and *S*_*j*_ is the corresponding SD.

Each protein can be translated into six vectors with each amino acid represented by normalized values of six descriptors. So, NMBAC [29] can be computed as follows:

10 $$ \begin{aligned} AC_{lag,j} &= \frac{1}{(n-lag)}\sum\limits_{i=1}^{n-lag}(X_{i,j} \times X_{i+lag,j})(i=1,2,\ldots,\\ &\qquad n-lag;j=1,2,\ldots,6.) \end{aligned}  $$

where *j* represents one descriptor of six descriptor, *i* is the position in protein sequence *X*, *n* is the length of the protein sequence and *lag* is the sequential distance between one residue and another, a certain number of residues away (*lag*=1,2,…,*lg*), and *lg* is a parameter determined by an optimization procedure to be described.

Inspired by AC [12], we select the optimal value of *lag* from 1 to 30. We can get 30×6=180 dimensional vector. We also compute the frequency of 20 amino acids appearing on this sequence. As a result, a protein sequence is represented as 30×6+20=200 features. Finally, we combine descriptors of two proteins, and build a 400-dimensional vector to represent each pair of proteins by NMBAC.

### Random forest classifier

RF is an algorithm for classification developed by Leo Breiman [31], which uses an ensemble of classification trees. Each classification tree is built by using a bootstrap sample of training data, and each split candidate set is a random subset of variables. RF uses both bagging (bootstrap aggregation) and random variable selection for tree building. Each classification tree is unpruned to obtain low-bias trees. The bagging and random variable selection can cause low correlation of individual trees. Therefore, RF has excellent performance in classification tasks.

In this paper, the feature space of each pair of proteins is composed of MMI and NMBAC. Totally, there are 238+400=638 features to be encoded to represent each pair of proteins. We define a 638-dimentional feature vector *F*=(*x*_1_,*x*_2_,…,*x*_638_) as the input data of RF model. The class label *t* of interacting pair or non-interacting pair is set as 1 or −1, respectively. If the number of cases in the training set is *N*, the sample is built by randomly choosing *N* cases from the original data, but with replacement. This sample will be the training set for growing the tree. There are *M* input variables, a number *m*≪*M* is specified such that at each node, *m* variables are selected at random out of *M* and the best split on these *m* is used to split the node. The value of *m* is held constant during the forest growing. Each tree is grown to the largest extent possible without pruning. For the new test sample, the classification result can be obtained by a voting method on these trees.

## Results

We test our method on several different PPIs datasets to evaluate the performance of our proposed approach, including *S*.*cerevisiae*,*H*.*pylori*,*human*_8161_,*C*.*elegans*,*E*.*coli*,*human*_1412_ and *M*.*musculus* dataset. First, we independently analyze the performance of two protein representations, such as MMI and NMBAC. Second, we compare our method with some outstanding methods on the *S*.*cerevisiae*,*H*.*pylori* and *human*_8161_ datasets. Then, we use the *S*.*cerevisiae* PPIs dataset to construct a model to predict other five independent species PPIs datasets. Our proposed method achieves a high performance on the *S*.*cerevisiae*,*H*.*pylori* and *human*_8161_ datasets, so we evaluate the prediction performance of our model on five independent testing datasets. Our experiments suggest that experimentally identified interactions in one organism are able to predict interactions in other organisms. We also test our method on two special yeast and human PPIs datasets. In addition, we test our method on three important PPIs networks, and compare it with the state-of-the-art methods. We use our primary experimental information to predict real PPIs network, which is assembled by pairwise PPIs data.

### PPIs datasets

The first PPIs dataset, described by You et al. [16], is downloaded from yeast *S*.*cerevisiae* core subset in the Database of Interacting Proteins (DIP) [11]. A protein with fewer than 50 residues or having more than 40 percent sequence identity are removed, and the remaining 5594 pairs of proteins formed the golden standard positive dataset (GSP). Non-interacting pairs are selected uniformly at random from the set of all interacting pairs that are not known to interact. Interacting pairs with the same subcellular localization information are then excluded. Finally, the golden standard negative dataset (GSN) is consisted of 5594 protein pairs, and their subcellular localization are different. The GSP and GSN datasets contain a total of 11188 protein pairs (half from the positive dataset and half from the negative dataset).

The second PPIs dataset, described by Martin et al. [32], is composed of 2916 *H*.*pylori* protein pairs (1458 interacting pairs and 1458 non-interacting pairs). The third PPIs dataset is collected from the Human Protein References Database (HPRD) as described by Huang et al. [19]. Huang et al. constructed the *human*_8161_ dataset by 8161 protein pairs (3899 interacting pairs and 4262 non-interacting pairs).

The *C*.*elegans*(4013 interacting pairs), *E*.*coli*(6954 interacting pairs), *human*_1412_(1412 interacting pairs), *M*.*musculus*(313 interacting pairs), and *H*.*pylori*(1420 interacting pairs) datasets are mentioned by Zhou et al. [14]. These species-specific PPIs datasets are employed in our experiment to verify the effectiveness of our proposed method.

### Evaluation measurements

To test the robustness of our method, we repeat the process of random selection of the training and test sets, model-building and model-evaluating. This process is five-fold cross validation. There are seven parameters: overall prediction accuracy (ACC), sensitivity (SN), specificity (Spec), positive predictive value (PPV), negative predictive value (NPV), weighted average of the PPV and sensitivity (F score), Matthew’s correlation coefficient (MCC). These parameters are defined as follows:

11a $$\begin{array}{*{20}l} ACC&=\frac{TP+TN}{TP+FP+TN+FN} \end{array} $$

11b $$\begin{array}{*{20}l} SN&=\frac{TP}{TP+FN} \end{array} $$

11c $$\begin{array}{*{20}l} Spec&=\frac{TN}{TN+FP} \end{array} $$

11d $$\begin{array}{*{20}l} PPV&=\frac{TP}{TP+FP} \end{array} $$

11e $$\begin{array}{*{20}l} NPV&=\frac{TN}{TN+FN} \end{array} $$

11f $$\begin{array}{*{20}l} F_{score}&=2 \times \frac{SN \times PPV}{SN + PPV} \end{array} $$

11g $${} \begin{aligned} MCC&=\frac{TP \times TN - FP \times FN}{\sqrt{(TP+FN)\times (TN+FP)\times (TP+FP) \times (TN+FN)}} \end{aligned}  $$

where true positive (TP) is the number of true PPIs that are predicted correctly; false negative (FN) is the number of true PPIs that are predicted to be non-interacting pairs; false positive(FP) is the number of true non-interacting pairs that are predicted to be PPIs, and true negative(TN) is the number of true non-interacting pairs that are predicted correctly.

### Experimental environment

In this paper, our proposed sequence-based PPIs predictor is implemented using C++ and MATLAB. All experiements are carried out on a computer with 2.5 GHz 6-core CPU, 32 GB memory and Windows operating system. Two RF parameters, the number of decision trees and split are 500 and 25.

### Performance of PPIs prediction

We use eight different datasets to evaluate the performance of our proposed method. The proposed approach is compared with other methods on the *S*.*cerevisiae*,*H*.*pylori* and *human*_8161_ datasets. Then, we test our method on the *human*_1412_,*M*.*musculus*,*H*.*pylori*,*C*.*elegans*, and *E*.*coli* datasets for PPIs prediction.

#### *S*.*cerevisiae* dataset

We use the first PPIs dataset used in You et al. [16] to evaluate the performance of our model.

##### Analyzing 2-tuples and 3-tuples MI

To analyze the performance of the 2-tuples and 3-tuples MI features by testing the *S*.*cerevisiae* dataset. The results of prediction for the 2-tuples and 3-tuples MI are shown in Table 3. The accuracies for 2-tuples MI, 3-tuples MI and MMI are 93.56,93.88 and 94.23 %, respectively. Obviously, the combinatorial approach of MMI achieves better performance than either 2-tuples MI or 3-tuples MI.

**Table 3:** Analyze the performance of 2-tuples and 3-tuples MI on *S*.*cerevisiae* dataset

Feature	Classifier	*ACC*(%)	*SN*(%)	*Spec*(%)	*PPV*(%)	*NPV*(%)	*F*1(%)	*MCC*(%)
2-tuples MI	RF	93.56 ±0.23	89.98 ±0.51	97.41 ±0.64	97.38 ±0.58	90.06 ±0.45	93.54 ±0.41	87.42 ±0.83
3-tuples MI	RF	93.88 ±0.25	90.25 ±0.42	97.30 ±0.50	96.94 ±0.44	91.35 ±0.55	93.47 ±0.39	87.92 ±0.77
MMI	RF	94.23 ±0.36	91.01 ±0.45	97.44 ±0.40	97.27 ±0.38	91.55 ±0.48	94.03 ±0.35	88.63 ±0.71

##### Selecting optimal *lag*

**Fig. 2:**
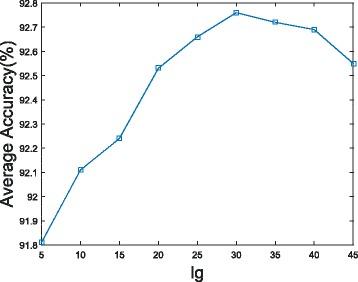
Accuracy of our method with NMBAC on different values of *lag*

The large value of *lag*=1,2,…,*lg* will result in more variables that account for residue contacts with large distances apart in the sequence. The maximal possible *lg* is the length of the shortest sequence (50 amino acids) in the dataset. To obtain the best *lg*, we test nine different values of *lg*(*lg*=5,10,15,20,25,30,35,40,45). The results of these nine values of *lg* on *S*.*cerevisiae* dataset are shown in Fig. 2. As seen from the curve, the prediction accuracy increases when *lg* increases from 5 to 30. However, it slightly declines when *lg* increases from 30 up to 45. The best prediction accuracy is 92.76 %, when *lg* is 30 amino acids. NMBAC with *lg* less than 30 would lose some useful features of protein sequences and larger values could introduce noise instead of improving the prediction performance. So, we select the optimal *lag* as 30 in our study.

##### Analyzing MMI and NMBAC

In order to understand the contribution of different feature representations, we evaluate the performance of MMI and NMBAC for PPIs prediction. We use the *S*.*cerevisiae* dataset, which is randomly partitioned into training and independent testing sets via a five-fold cross validation. Each of the five subsets acts as an independent holdout testing dataset for the model trained with rest four subsets. The cross validation can minimize the impact of data dependency and the reliability of experimental results can be improved. The prediction result is showed in Table 4. The accuracies for MMI, NMBAC and ensemble representation are 94.23,92.76 and 95.01 %, respectively. Obviously, MMI has better performance than NMBAC. Using ensemble representation, accuracy can be raised 0.78 %.

**Table 4:** Analyze the performance of MMI and NMBAC on *S*.*cerevisiae* dataset by RF Classifier

Feature	*ACC*(%)	*SN*(%)	*Spec*(%)	*PPV*(%)	*NPV*(%)	*F*1(%)	*MCC*(%)
MMI	94.23 ±0.36	91.01 ±0.45	97.44 ±0.40	97.27 ±0.38	91.55 ±0.48	94.03 ±0.35	88.63 ±0.71
NMBAC	92.76 ±0.35	90.99 ±0.59	94.53 ±0.50	94.34 ±0.37	91.30 ±0.68	92.63 ±0.26	85.57 ±0.70
MMI+NMBAC(A-B order)	95.01 ±0.46	92.67 ±0.50	97.31 ±0.61	97.16 ±0.55	93.06 ±0.48	94.26 ±1.18	90.10 ±0.92
MMI+NMBAC(B-A order)	94.90 ±0.24	92.60 ±0.47	97.22 ±0.58	97.10 ±0.44	92.89 ±0.55	94.79 ±0.78	89.91 ±1.1

To consider the asymmetric of proteins, the forward vector of one PPI is composed of two interacting proteins (protein A and protein B), and the backward vector is composed of reverse two interacting proteins (protein B and protein A). Accuracies on forward and backward vectors for PPIs prediction are 95.01 and 94.90 %, and the prediction result is less changed.

##### 5-fold cross-validation

The prediction result of our method on *S*.*cerevisiae* dataset is shown in Table 5. We predict PPIs of *S*.*cerevisiae* dataset, and obtain accuracy, precision, sensitivity, and MCC of 95.01,97.31,92.67, and 90.1 %, respectively. Standard deviations of these criteria values are 0.46,0.61,0.5, and 0.92 %, respectively. High accuracies and low standard deviations of these criterion values show that our proposed model is effective and stable for predicting PPIs.

**Table 5:** 5-fold cross-validation result obtained by using our proposed method on *S*.*cerevisiae* dataset

Testing set	*ACC*(%)	*SN*(%)	*Spec*(%)	*PPV*(%)	*NPV*(%)	*F*1(%)	*MCC*(%)
1	95.41	93.15	97.60	97.46	93.54	92.26	90.88
2	94.99	92.03	97.82	97.57	92.80	94.72	90.11
3	94.28	92.31	96.29	96.23	92.44	94.23	88.64
4	94.95	92.69	97.22	97.10	92.97	94.84	89.99
5	95.40	93.15	97.60	97.46	93.54	95.26	90.88
Average	95.01 ±0.46	92.67 ±0.5	97.31 ±0.61	97.16 ±0.55	93.06 ±0.48	94.26 ±1.18	90.1 ±0.92

##### Comparison with existing methods

We compare the prediction performance of our proposed method with other existing methods on the *S*.*cerevisiae* dataset, as showed in Table 6. It can be observed that high prediction accuracy of 95.01 % is obtained from our proposed model. We use the same *S*.*cerevisiae* PPIs dataset, and compare our experimental result with methods proposed by You et al. [16, 18, 30], Wong et al. [17], Guo et al. [12], Zhou et al. [14] and Yang et al. [15], where Random Forest (RF), Ensemble Extreme Learning Machines (EELM), Support Vector Machine (SVM), Rotation Forest, Support Vector Machine (SVM), or k-Nearest Neighbor (KNN) is performed with MLD, AC +*CT*+*LD*+MAC, MCD, PR-LPQ, AC, ACC, or LD scheme as input feature vectors, respectively. Their prediction accuracies are 94.72±0.43,87.00±0.29,91.36±0.36,93.92±0.36,89.33±2.67,87.36±1.38,88.56±0.33, and 86.15±1.17 %, respectively, whereas our prediction accuracy is 95.01±0.46 %. Our method has the highest prediction accuracy on the *S*.*cerevisiae* PPIs dataset, compared to all above methods. Our method has the best performance in other criteria as well. The sensitivity is 92.67±0.5 %, and the Matthew’s correlation coefficient is 90.1±0.92 % in our result. On the *S*.*cerevisiae* dataset, the MCC of our method is better than other existing methods.

**Table 6:** Comparison of the prediction performance between our proposed method and other state-of-the-art works on *S*.*cerevisiae* dataset

Method	Feature	Classifier	*ACC*(%)	*SN*(%)	*PPV*(%)	*MCC*(%)
Our method	MMI+NMBAC	RF	95.01 ±0.46	92.67 ±0.50	97.16 ±0.55	90.10 ±0.92
You’s work [18]	MLD	RF	94.72 ±0.43	94.34 ±0.49	98.91 ±0.33	85.99 ±0.89
You’s work [30]	AC+CT+LD+MAC	E-ELM	87.00 ±0.29	86.15 ±0.43	87.59 ±0.32	77.36 ±0.44
You’s work [16]	MCD	SVM	91.36 ±0.36	90.67 ±0.69	91.94 ±0.62	84.21 ±0.59
Wong’s work [17]	PR-LPQ	Rotation Forest	93.92 ±0.36	91.10 ±0.31	96.45 ±0.45	88.56 ±0.63
Guo’s work [12]	ACC	SVM	89.33 ±2.67	89.93 ±3.68	88.87 ±6.16	N/A ^a^
Guo’s work [12]	AC	SVM	87.36 ±1.38	87.30 ±4.68	87.82 ±4.33	N/A ^a^
Zhou’s work [14]	LD	SVM	88.56 ±0.33	87.37 ±0.22	89.50 ±0.60	77.15 ±0.68
Yang’s work [15]	LD	KNN	86.15 ±1.17	81.03 ±1.74	90.24 ±1.34	N/A ^a^

^a^N/A means not available

#### *H*.*pylori* dataset

In order to highlight the advantage of our method, we also test it on the *H*.*pylori* dataset, which is described by Martin et al. [32]. We compare the prediction performance of our proposed method with other previous works including AC+CT+LD+MAC [30], MCD [16] DCT + SMR [19], phylogenetic bootstrap [33], signature products [32], HKNN [24], ensemble of HKNN [25] and boosting. In Table 7, we can see that the average prediction performance of our method, such as sensitivity, PPV, accuracy and MCC are 87.59,86.81,88.23 and 75.24 %, respectively. On the *H*.*pylori* dataset, the accuracy of our method is better than all other methods tested. It is shown that our method deeply extracts the contiguous amino acid information from protein sequence. Furthermore, our method combining MMI and NMBAC can increase the prediction performance. The accuracies for MMI, NMBAC and ensemble representation are 85.42,85.59 and 87.59 %, respectively. The accuracy can be increased by at least 2.00 % on the *H*.*pylori* dataset.

**Table 7:** Comparison of the prediction performance between our proposed method and other different methods on *H*.*pylori* dataset

Methods	*ACC*(%)	*SN*(%)	*PPV*(%)	*MCC*(%)
Our method(MMI + NMBAC)	87.59	86.81	88.23	75.24
Our method(MMI)	85.42	85.22	87.70	70.71
Our method(NMBAC)	85.59	83.33	89.53	71.35
You’s work(AC+CT+LD+MAC) [30]	87.50	88.95	86.15	78.13
You’s work(MCD)[16]	84.91	83.24	86.12	74.40
Huang’s work(DCT + SMR) [19]	86.74	86.43	87.01	76.99
Phylogenetic bootstrap [33]	75.80	69.80	80.20	N/A ^a^
HKNN [24]	84.00	86.00	84.00	N/A ^a^
Signature products [32]	83.40	79.90	85.70	N/A ^a^
Ensemble of HKNN [25]	86.60	86.70	85.00	N/A ^a^
Boosting	79.52	80.37	81.69	70.64

^a^N/A means not available

#### *human*_8161_ dataset

We also test our method on a *human*_8161_ dataset, which is used by Huang et al. [19]. We compare the prediction performance between our proposed method and Huang’s work [19] on this dataset, as showed in Table 8. Our method achieves 97.56 % accuracy, 96.57 % sensitivity and 95.13 % MCC. However, Huang’s work achieved 96.30 % accuracy, 92.63 % sensitivity and 92.82 % MCC. Our method obtains better prediction result than Huang’s work on *human*_8161_ dataset. Particularly, accuracies for MMI, NMBAC and ensemble representation are 97.56,96.08 and 95.59 %, respectively. The accuracy can be raised 1.48 % on *human*_8161_ dataset.

**Table 8:** Comparison of the prediction performance between our proposed method and other different methods on *human*_8161_ dataset

Methods	*ACC*(%)	*SN*(%)	*PPV*(%)	*MCC*(%)
Our method(MMI + NMBAC)	97.56	96.57	98.30	95.13
Our method(MMI)	96.08	95.05	96.97	92.17
Our method(NMBAC)	95.59	94.06	96.94	91.21
Huang’s work(DCT + SMR) [19]	96.30	92.63	99.59	92.82

### PPIs identification on independent across species dataset

If large number of physically interacting proteins in one organism exist “co-evolved” relationship, their respective orthologs in other organisms interact as well. In this section, we use all 11,188 samples of the *S*.*cerevisiae* dataset as the training set and other species datasets (*E*.*coli*,*C*.*elegans*,*human*_1412_,*H*.*pylori* and *M*.*musculus*) as the test sets. The performance of these five experiments is summarized in Table 9. The accuracies are 92.80,92.16,94.33,91.13, and 95.85 % on the *E*.*coli*,*C*.*elegans*,*human*_1412_,*H*.*pylori* and *M*.*musculus* datasets, respectively. The result of our method is better than other methods [14, 18, 19]. Overall, the accuracy of ensemble representation is raised by 2.79 % than single representation (MMI and NMBAC) on these five independent species.

**Table 9:** Prediction results on five independent species by our proposed method, based on *S*.*cerevisiae* dataset as the training set

Species	Testing pairs	ACC(%)					
		MMI + NMBAC	MMI	NMBAC	You’s work [18]	Huang’s work [19]	Zhou’s work [14]
*E*.*coli*	6954	92.80	89.01	90.13	89.30	66.08	71.24
*C*.*elegans*	4013	92.16	88.54	86.72	87.71	81.19	75.73
*human*_1412_	1412	94.33	91.31	90.23	94.19	82.22	76.27
*H*.*pylori*	1420	91.13	90.28	90.34	90.99	82.18	N/A ^a^
*M*.*musculus*	313	95.85	92.01	91.37	91.96	79.87	76.68

^a^N/A means not available

### Two special PPIs datasets

Yungki Park and Edward M. Marcotte [20] proposed two PPIs datasets to evaluate pair-input computational predictions, including *yeast* and *human* data sets. We compare the performance of our method with seven methods (*M*_1_−*M*_7_) of pair-input computational predictions on the two PPIs datasets: *M*_1_, a signature products-based method proposed by Martin et al. [32] and classified by SVM; *M*_2_, a protein sequence is described as in *M*_1_, and the feature vector for a protein pair is formed by applying the metric learning pairwise kernel and classified by SVM; *M*_3_, the SVM-based method of CT feature developed by Shen et al. [13]; *M*_4_, the SVM-based method of AC feature developed by Guo et al. [12]; *M*_5_, the PPIs feature is same as *M*_4_, and the classifier is the random forest; *M*_6_, a method developed by Pitre et al. [34]; *M*_7_, a method originally developed for protein-RNA interaction prediction [35]. We use the typical cross-validated (CV) predictive performances for three distinct test classes (*C*1,*C*2,*C*3). The performance of each method is summarized as the average area under the receiver operating characteristic curve (AUROC) ± its standard deviation and the corresponding average area under the precision-recall curve (AUPRC) ± its standard deviation.

Prediction results are shown in Tables 10 and 11. On the *yeast* PPIs dataset, our method achieves 0.82,0.82,0.62 and 0.61 AUROC values on *CV*,*C*1,*C*2, and *C*3, respectively. Moreover, AUROC values on *CV*,*C*1,*C*2, and *C*3 are 0.82,0.82,0.60 and 0.57 on the *human* dataset, respectively. Our method obtains better prediction result than *M*_1_−*M*_7_ on *yeast* and *human* datasets.

**Table 10:** Comparison of prediction performance between our proposed method and other seven methods on the *yeast* dataset

Method	AUROC				AUPRC			
	CV	C1	C2	C3	CV	C1	C2	C3
MMI+NMBAC	0.82 ±0.02	0.82 ±0.01	0.62 ±0.02	0.61 ±0.02	0.84 ±0.01	0.84 ±0.01	0.64 ±0.02	0.62 ±0.02
MMI	0.82 ±0.01	0.82 ±0.01	0.62 ±0.02	0.60 ±0.02	0.84 ±0.02	0.84 ±0.01	0.64 ±0.02	0.61 ±0.02
NMBAC	0.82 ±0.01	0.82 ±0.01	0.61 ±0.02	0.60 ±0.03	0.83 ±0.01	0.83 ±0.01	0.63 ±0.03	0.60 ±0.03
M1	0.82 ±0.01	0.82 ±0.01	0.61 ±0.02	0.58 ±0.03	0.83 ±0.02	0.83 ±0.01	0.62 ±0.02	0.57 ±0.03
M2	0.83 ±0.01	0.84 ±0.01	0.60 ±0.02	0.59 ±0.03	0.84 ±0.02	0.84 ±0.01	0.61 ±0.02	0.58 ±0.03
M3	0.61 ±0.01	0.61 ±0.01	0.53 ±0.01	0.50 ±0.01	0.65 ±0.02	0.65 ±0.02	0.56 ±0.03	0.53 ±0.07
M4	0.76 ±0.02	0.76 ±0.02	0.57 ±0.02	0.54 ±0.03	0.76 ±0.02	0.76 ±0.02	0.58 ±0.02	0.54 ±0.03
M5	0.80 ±0.02	0.80 ±0.01	0.58 ±0.01	0.55 ±0.02	0.78 ±0.02	0.78 ±0.01	0.57 ±0.02	0.54 ±0.02
M6	0.75 ±0.02	0.75 ±0.02	0.59 ±0.04	0.52 ±0.04	0.75 ±0.02	0.76 ±0.02	0.60 ±0.05	0.47 ±0.07
M7	0.58 ±0.02	0.58 ±0.01	0.54 ±0.02	0.52 ±0.03	0.60 ±0.02	0.60 ±0.02	0.55 ±0.02	0.53 ±0.02

**Table 11:** Comparison of prediction performance between our proposed method and other seven methods on the *human* dataset

Method	AUROC				AUPRC			
	CV	C1	C2	C3	CV	C1	C2	C3
MMI+NMBAC	0.82 ±0.01	0.82 ±0.01	0.60 ±0.01	0.57 ±0.02	0.83 ±0.01	0.83 ±0.01	0.60 ±0.01	0.56 ±0.02
MMI	0.81 ±0.01	0.81 ±0.01	0.59 ±0.01	0.56 ±0.02	0.82 ±0.01	0.83 ±0.01	0.59 ±0.01	0.55 ±0.01
NMBAC	0.81 ±0.01	0.82 ±0.01	0.60 ±0.01	0.57 ±0.02	0.83 ±0.01	0.83 ±0.01	0.60 ±0.01	0.56 ±0.02
M1	0.81 ±0.01	0.81 ±0.01	0.61 ±0.01	0.58 ±0.03	0.82 ±0.01	0.82 ±0.01	0.60 ±0.01	0.57 ±0.03
M2	0.85 ±0.01	0.85 ±0.01	0.60 ±0.01	0.58 ±0.02	0.85 ±0.00	0.85 ±0.01	0.60 ±0.01	0.56 ±0.02
M3	0.63 ±0.01	0.64 ±0.01	0.55 ±0.01	0.50 ±0.00	0.67 ±0.01	0.67 ±0.01	0.57 ±0.02	0.52 ±0.05
M4	0.77 ±0.01	0.77 ±0.01	0.57 ±0.02	0.53 ±0.02	0.77 ±0.01	0.77 ±0.01	0.56 ±0.01	0.53 ±0.02
M5	0.81 ±0.01	0.81 ±0.01	0.59 ±0.01	0.54 ±0.02	0.82 ±0.01	0.82 ±0.01	0.59 ±0.01	0.54 ±0.02
M6	0.76 ±0.01	0.77 ±0.01	0.64 ±0.01	0.59 ±0.02	0.79 ±0.01	0.79 ±0.01	0.67 ±0.01	0.59 ±0.02
M7	0.56 ±0.01	0.56 ±0.01	0.53 ±0.01	0.54 ±0.02	0.56 ±0.01	0.56 ±0.01	0.53 ±0.01	0.54 ±0.02

Yungki Park and Edward M. Marcotte [20] also constructed new *yeast* and *human* PPIs datasets by suppressing the representational bias-driven learning. Prediction results are shown in Table 12 and Table 13. On new *yeast* PPIs dataset, our method achieves 0.65,0.66,0.60 and 0.55 AUROC on *CV*,*C*1,*C*2, and *C*3, respectively. On average, our method obtains better prediction result than *M*_1_−*M*_7_ on new *yeast* dataset. On new *human* dataset, our proposed method achieves 0.61,0.62,0.57 and 0.53 AUROC on *CV*,*C*1,*C*2, and *C*3, respectively. On average, our result is also better than *M*_2_−*M*_7_, but does not outperform *M*_1_ on the new *human* dataset.

**Table 12:** Comparison of prediction performance between our proposed method and other seven methods on new *yeast* dataset, suppressing representation bias-driven learning

Method	AUROC				AUPRC			
	CV	C1	C2	C3	CV	C1	C2	C3
MMI+NMBAC	0.65 ±0.02	0.66 ±0.02	0.60 ±0.02	0.55 ±0.02	0.67 ±0.02	0.68 ±0.02	0.60 ±0.02	0.55 ±0.02
MMI	0.64 ±0.02	0.65 ±0.01	0.60 ±0.02	0.55 ±0.02	0.66 ±0.02	0.68 ±0.01	0.60 ±0.02	0.54 ±0.02
NMBAC	0.63 ±0.02	0.64 ±0.02	0.59 ±0.02	0.54 ±0.03	0.65 ±0.02	0.66 ±0.02	0.59 ±0.02	0.54 ±0.02
M1	0.64 ±0.01	0.64 ±0.01	0.62 ±0.02	0.57 ±0.04	0.65 ±0.01	0.65 ±0.01	0.61 ±0.02	0.56 ±0.03
M2	0.61 ±0.01	0.61 ±0.02	0.62 ±0.02	0.58 ±0.03	0.61 ±0.01	0.61 ±0.02	0.62 ±0.02	0.57 ±0.03
M3	0.54 ±0.01	0.55 ±0.01	0.53 ±0.01	0.50 ±0.01	0.60 ±0.02	0.60 ±0.01	0.56 ±0.03	0.53 ±0.07
M4	0.55 ±0.02	0.55 ±0.02	0.54 ±0.02	0.51 ±0.02	0.53 ±0.02	0.53 ±0.01	0.53 ±0.02	0.51 ±0.02
M5	0.60 ±0.02	0.60 ±0.01	0.55 ±0.02	0.52 ±0.02	0.61 ±0.02	0.61 ±0.01	0.55 ±0.02	0.51 ±0.02
M7	0.55 ±0.02	0.54 ±0.01	0.54 ±0.02	0.53 ±0.03	0.55 ±0.02	0.55 ±0.01	0.54 ±0.02	0.53 ±0.02

**Table 13:** Comparison of prediction performance between our proposed method and other seven methods on new *human* dataset, suppressing representation bias-driven learning

Method	AUROC				AUPRC			
	CV	C1	C2	C3	CV	C1	C2	C3
MMI+NMBAC	0.61 ±0.01	0.62 ±0.01	0.57 ±0.02	0.53 ±0.01	0.64 ±0.01	0.65 ±0.01	0.58 ±0.02	0.53 ±0.01
MMI	0.61 ±0.01	0.62 ±0.01	0.57 ±0.01	0.53 ±0.01	0.64 ±0.01	0.65 ±0.01	0.58 ±0.01	0.53 ±0.01
NMBAC	0.59 ±0.01	0.60 ±0.01	0.56 ±0.01	0.52 ±0.02	0.62 ±0.01	0.63 ±0.01	0.56 ±0.01	0.52 ±0.01
M1	0.64 ±0.01	0.65 ±0.01	0.61 ±0.01	0.57 ±0.02	0.66 ±0.01	0.67 ±0.01	0.61 ±0.02	0.56 ±0.02
M2	0.59 ±0.01	0.60 ±0.01	0.60 ±0.01	0.57 ±0.02	0.60 ±0.01	0.61 ±0.01	0.59 ±0.01	0.55 ±0.01
M3	0.54 ±0.01	0.55 ±0.01	0.53 ±0.01	0.50 ±0.00	0.61 ±0.01	0.61 ±0.01	0.56 ±0.02	0.52 ±0.05
M4	0.56 ±0.01	0.56 ±0.01	0.54 ±0.01	0.52 ±0.02	0.54 ±0.01	0.54 ±0.01	0.53 ±0.01	0.52 ±0.01
M5	0.59 ±0.01	0.60 ±0.01	0.56 ±0.01	0.53 ±0.01	0.63 ±0.01	0.64 ±0.01	0.57 ±0.01	0.53 ±0.01
M7	0.55 ±0.01	0.55 ±0.01	0.53 ±0.01	0.53 ±0.03	0.55 ±0.01	0.55 ±0.01	0.53 ±0.01	0.54 ±0.02

### PPIs networks prediction

**Fig. 3:**
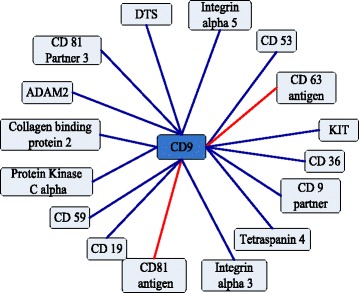
An one-core network for the CD9 network

The useful application of PPIs prediction method is the capability of predicting PPIs networks. Our method predicts three important PPI networks assembled by PPIs pairwise. The one-core network of CD9 is the simplest network, which is an important tetraspanin protein [21]. The result reveals that 14 of all 16 PPIs could be identified by our method, and accuracy is 87.50 %. Comparing to Shen’s work [13], accuracy of our method is raised 6.25 %. Results are shown in Fig. 3, and the dark blue lines are true prediction, and red lines are false prediction.

**Fig. 4:**
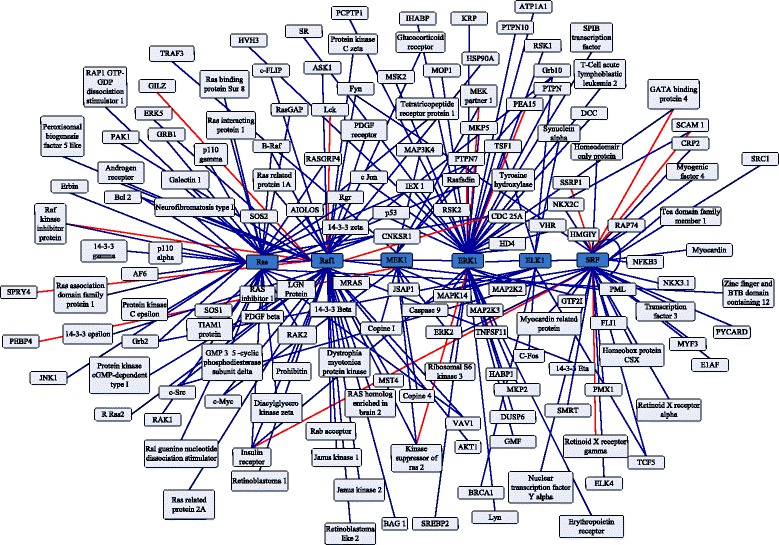
A multiple-cores network for the Ras-Raf-Mek-Erk-Elk-Srf pathway

The Ras-Raf-Mek-Erk-Elk-Srf pathway is a multiple-core network that has been implicated in a variety of cellular processes [22]. There are 189 PPIs in this network, 174 PPIs are predicted correctly by our method. Comparing to Shen’s work, accuracy is raised 2.06 %. The prediction result and Ras-Raf-Mek-Erk-Elk-Srf pathway are shown in Fig. 4. The dark blue lines are true prediction, and red lines are false prediction.

**Fig. 5:**
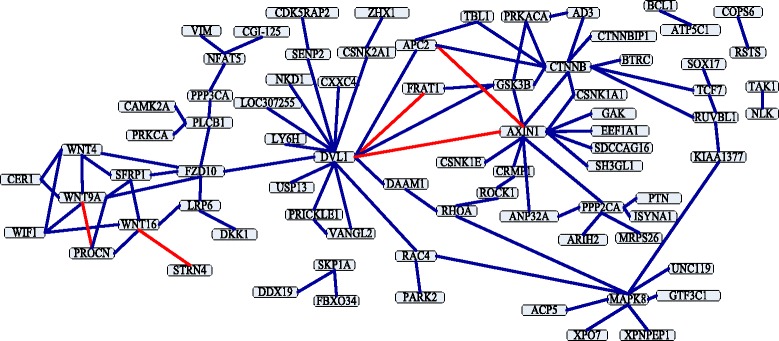
A crossover network for the Wnt-related pathway

The Wnt-related network is a typical crossover network, and its related pathway is essential in signal transduction. Ulrich et al. [23] has demonstrated the protein interaction topology of Wnt-related network. Shen et al. [13] have tested their method on the network. The accuracy of their method is 76.04 % in the network: there are 96 PPIs in this network, and 73 PPIs are predicted correctly by their method. We also try to predict PPIs in the Wnt-related network. The prediction result shows that 91 PPIs among all 96 PPIs in the network are discovered by our method, and the accuracy is 94.79 %, which is better than Shen’s method [13]. The prediction result and Wnt-related network are shown in Fig. 5. The dark blue lines are true prediction, and red lines are false prediction.

## Discussion

Although many computational methods have been used to predict PPIs, the effectiveness of previous prediction models can still be improved. Existing methods that fail to take into account local amino acid environments are neither reliable nor robust, therefore we propose a Conjoint Triad method that accounts for the properties of each amino acid when accompanied by its two vicinal peptide amino acids.

We use one PPIs dataset to construct a model to predict other five independent species PPIs datasets. This finding indicates that the proposed model can be successfully applied to other species for which experimental PPIs data is not available. It should be noticed that the biological hypothesis of mapping PPIs from one species to another species is that large numbers of physically interacting proteins in one organism are co-evolved.

The most useful application of PPIs prediction method is its capability of predicting PPIs networks. Accurately predicting PPI networks is the most important issue for PPI prediction methods. We extend our method to predict three real important PPIs networks: one-core network, multiple-core network and crossover network. General PPIs networks are crossover networks, so our method is useful in practical applications. All these results demonstrate that our proposed method is a very promising and useful support tool for future proteomics research. Main improvements of the proposed method come from adopting an effective feature extraction method that can capture useful protein sequence information. In the future work, we will extend our method to predict other important PPIs networks.

## Conclusions

In this paper, we develop a new method for predicting PPIs using primary sequences of two proteins. The prediction model is constructed based on random forest and ensemble feature representation scheme. In addition, we use MMI to improve the performance in predicting PPIs. For the performance evaluation, our method is applied to *S*.*cerevisiae* PPIs dataset. The prediction result shows that our method achieves 95.01 % accuracy and 92.67 % sensitivity. To further demonstrating the effectiveness of our method, we also use *H*.*pylori* PPIs dataset. Our method achieves 87.59 % accuracy and 86.81 % sensitivity. On *human*_8161_ dataset, the experimental result shows that our method achieves 97.56 % accuracy and 96.57 % sensitivity. We use *S*.*cerevisiae* PPIs dataset to construct a model to predict other five independent species PPIs datasets. Our proposed method achieves 92.80,92.16,94.33,91.13, and 95.85 % accuracies on *E*.*coli*,*C*.*elegans*,*human*_1412_,*H*.*pylori* and *M*.*musculus* datasets, respectively. We extend our method to predict three real important PPIs networks, and accuracy of our method is increased 6.25,2.06 and 18.75 % compared with CT. The prediction ability of our approach is better than that of other existing PPIs prediction methods.
